# Supplementary material for: Gemcitabine-Vitamin E Prodrug-Loaded Micelles for Pancreatic Cancer Therapy
Source: Pharmaceutics. 2024 Jan 10;16(1):95. doi: 10.3390/pharmaceutics16010095 (PMC10819901; doi:10.3390/pharmaceutics16010095)
Supplement: Supplementary file 1 [file pharmaceutics-16-00095-s001.zip › pharmaceutics-2794284-supplementary.pdf]

SUPPLEMENTARY MATERIAL

Gemcitabine-Vitamin E Prodrug-Loaded Micelles for Pancreatic Cancer Therapy

Miguel Pereira-Silva [1,2,3], Darío Miranda-Pastoriza [4,5], Luis Diaz-Gomez [3], Eddy Sotelo [4,5], Ana Cláudia Paiva-Santos [1,2], Francisco Veiga [1,2], Angel Concheiro [3,*] and Carmen Alvarez-Lorenzo [3,*]

[1] Department of Pharmaceutical Technology, Faculty of Pharmacy of the University of Coimbra, University of Coimbra, 3000-548 Coimbra, Portugal
[2] REQUIMTE/LAQV, Group of Pharmaceutical Technology, Faculty of Pharmacy of the University of Coimbra, University of Coimbra, 3000-548 Coimbra, Portugal
[3] Departamento de Farmacología, Farmacia y Tecnología Farmacéutica, I+D Farma (GI-1645), Facultad de Farmacia, Instituto de Materiales (iMATUS) and Health Research Institute of Santiago de Compostela (IDIS), Universidade de Santiago de Compostela, 15782 Santiago de Compostela, Spain
[4] Department of Organic Chemistry, Faculty of Farmacy, Universidade de Santiago de Compostela, 15782 Santiago de Compostela, Spain
[5] Centro Singular de Investigación en Química Biolóxica e Materiais Moleculares (CIQUS), Universidade de Santiago de Compostela, 15782 Santiago de Compostela, Spain
***** Correspondence: angel.concheiro@usc.es (A.C.); carmen.alvarez.lorenzo@usc.es (C.A.-L.); Tel.: +34-881815239 (C.A.-L.)

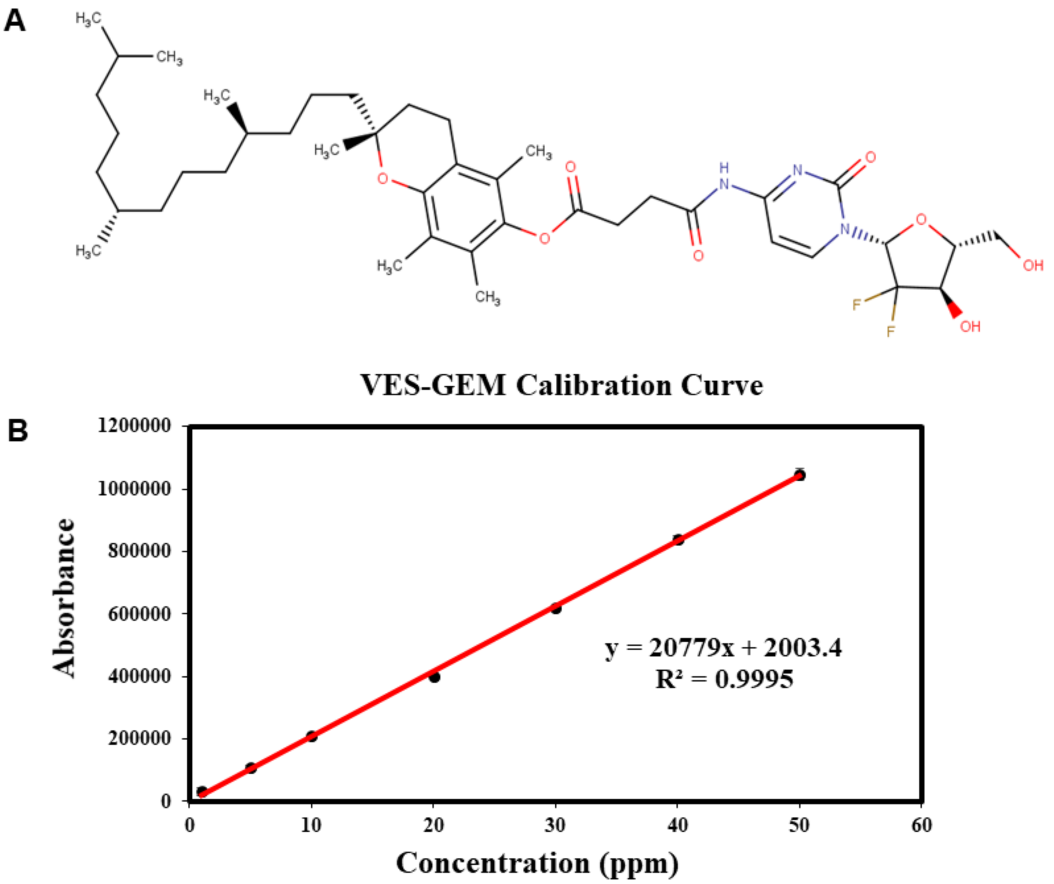

Figure S1. (A) Chemical structure of VES-GEM conjugate, Chemicalize and MarvinView software models (Chemicalize.com; ChemAxon Ltd., Budapest, Hungary). (B) Calibration curve for HPLC quantification of VES-GEM conjugate. Typical chromatograms are shown in **Figure S2** and **Figure S3**.

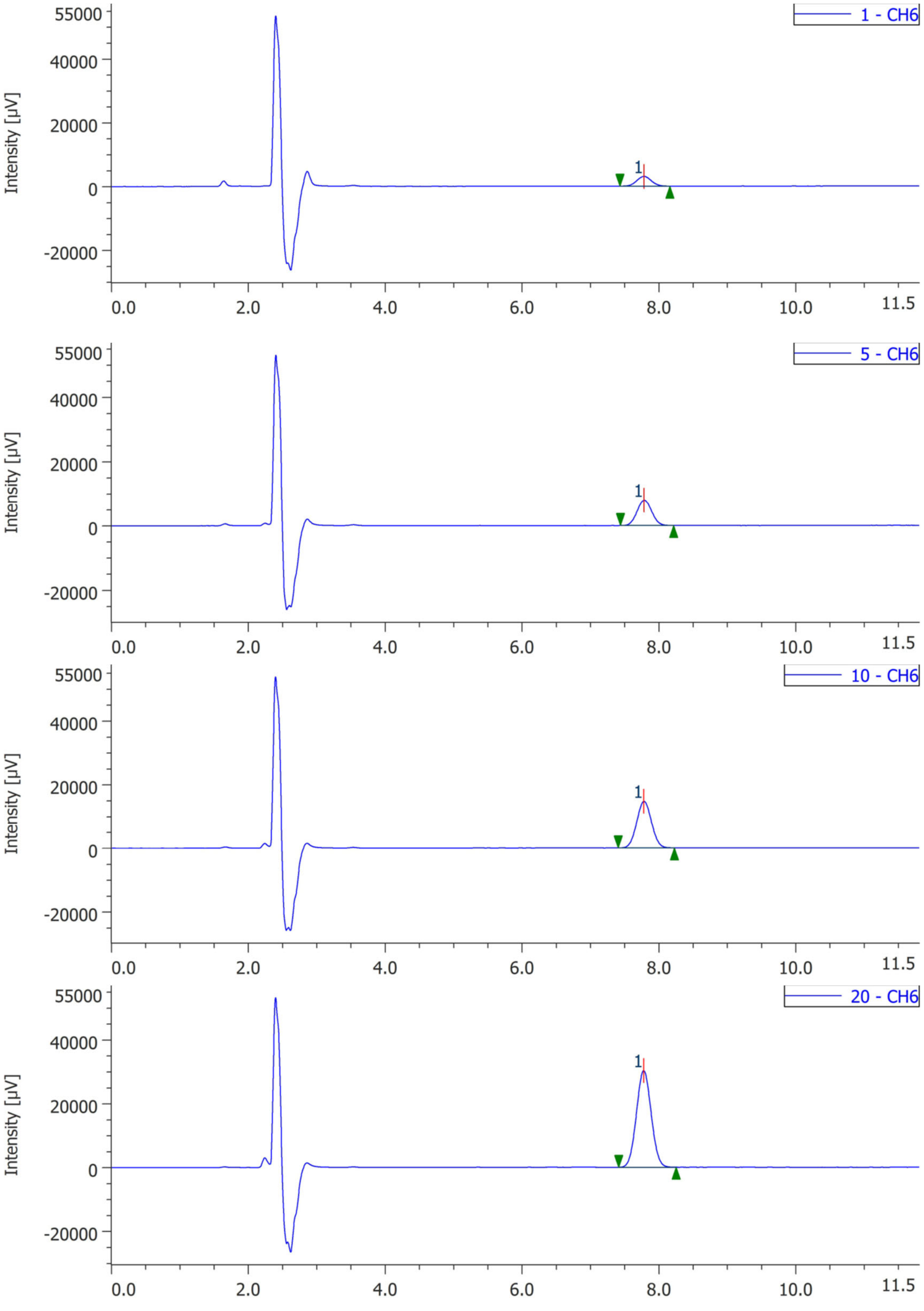
1 - CH6
5 - CH6
10 - CH6
20 - CH6
Intensity [µV]
55000
40000
20000
0
-20000
0.0
2.0
4.0
6.0
8.0
10.0
11.5
1

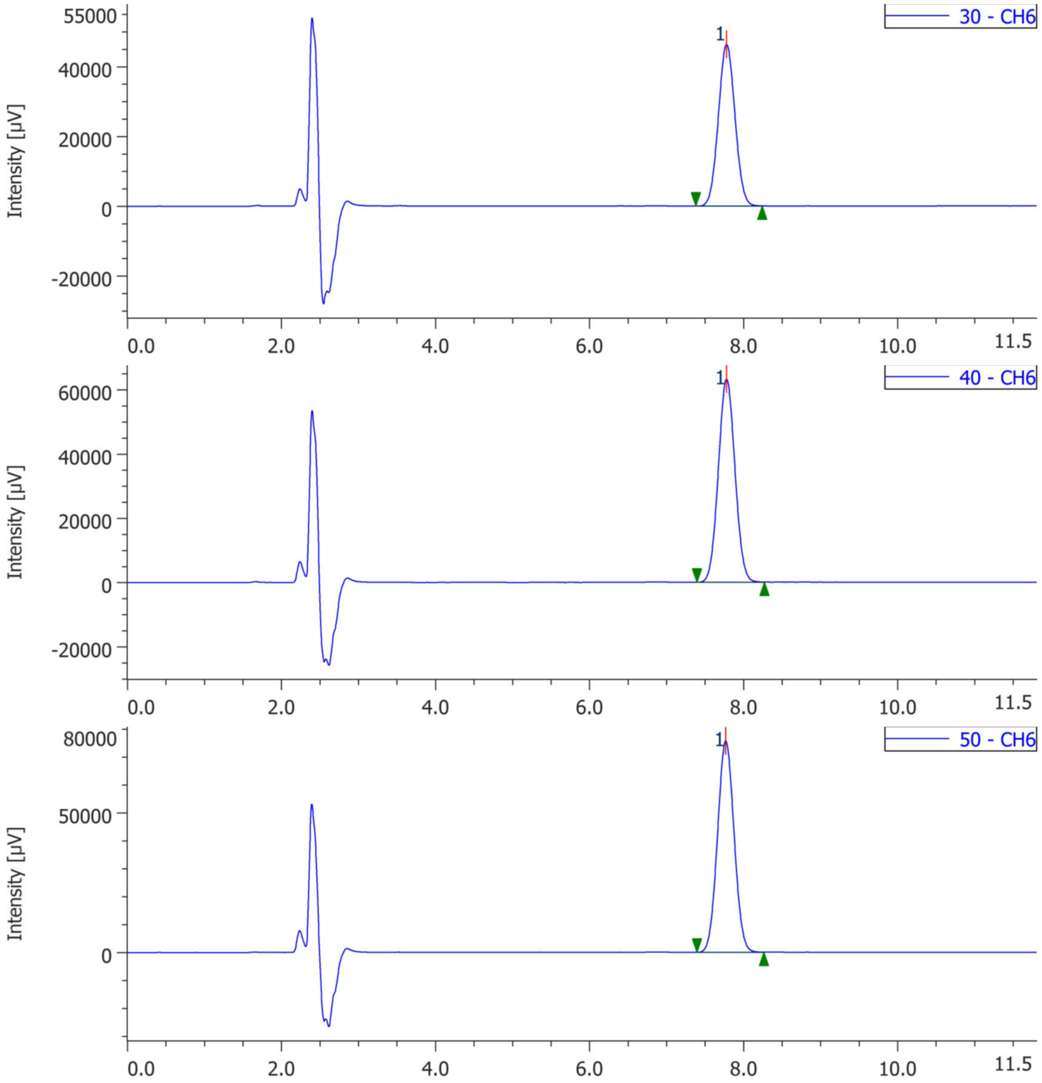

Figure S2. Typical chromatograms obtained for building VES-GEM calibration curve, R.T. ~8 min.

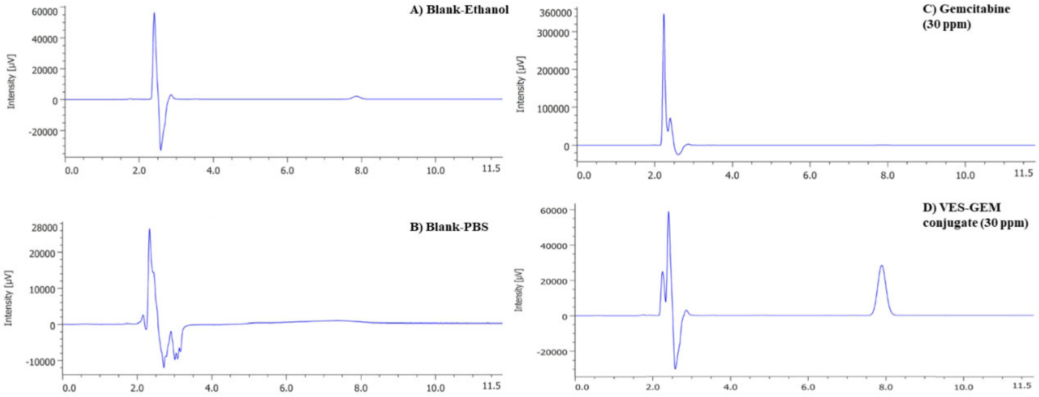

Figure S3. Chromatograms of blanks (ethanol or PBS), gemcitabine (30 ppm) and VES-GEM conjugate (30 ppm). The later (D) can be detected using the reported HPLC method.

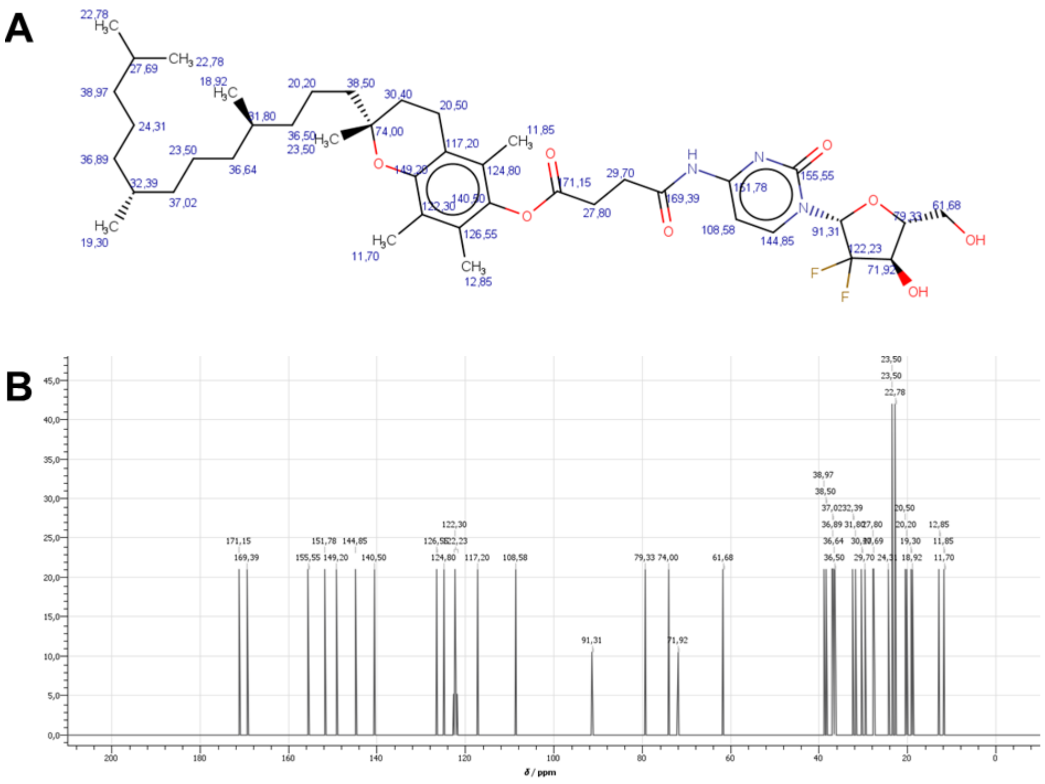

Figure S4. (A-B) Predicted [13] C NMR spectrum of VES-GEM conjugate. Chemicalize and MarvinView software models (Chemicalize.com; ChemAxon Ltd., Budapest, Hungary).

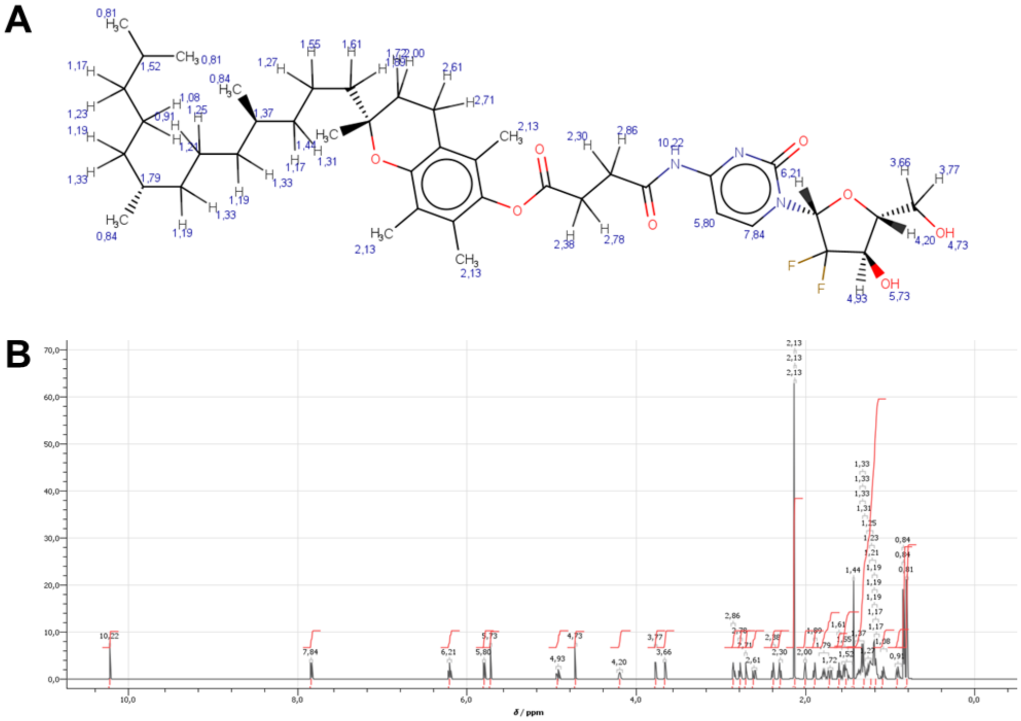

Figure S5. (A-B) Predicted [1] H NMR spectrum of VES-GEM conjugate. Chemicalize and MarvinView software models.

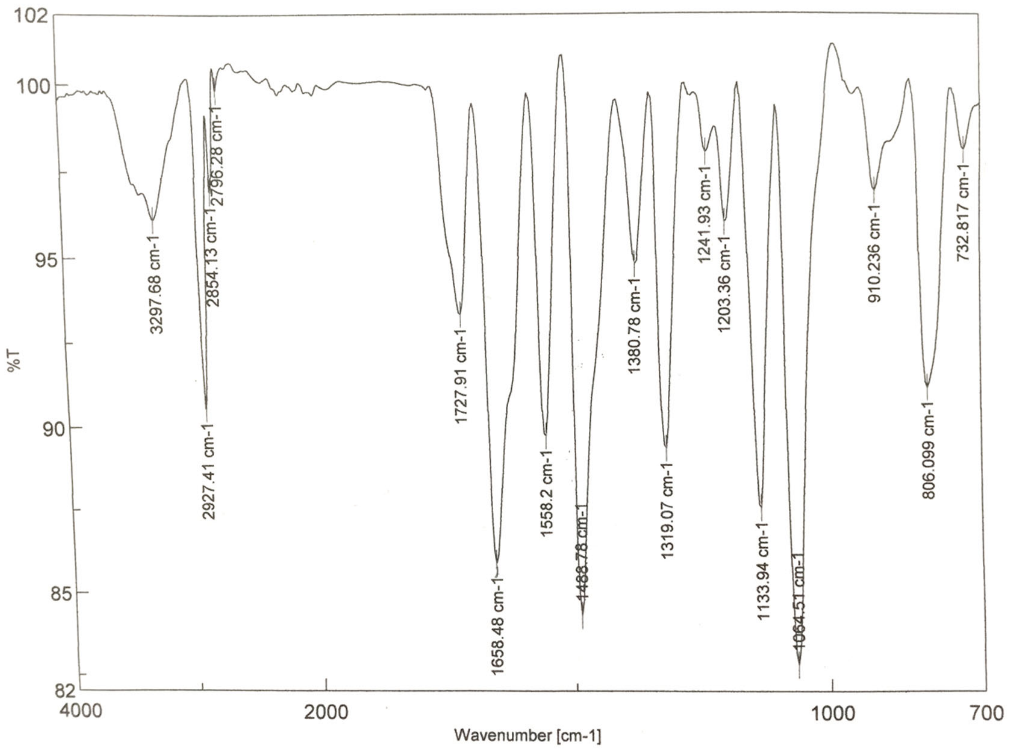

Figure S6. FTIR spectra of VES-GEM.

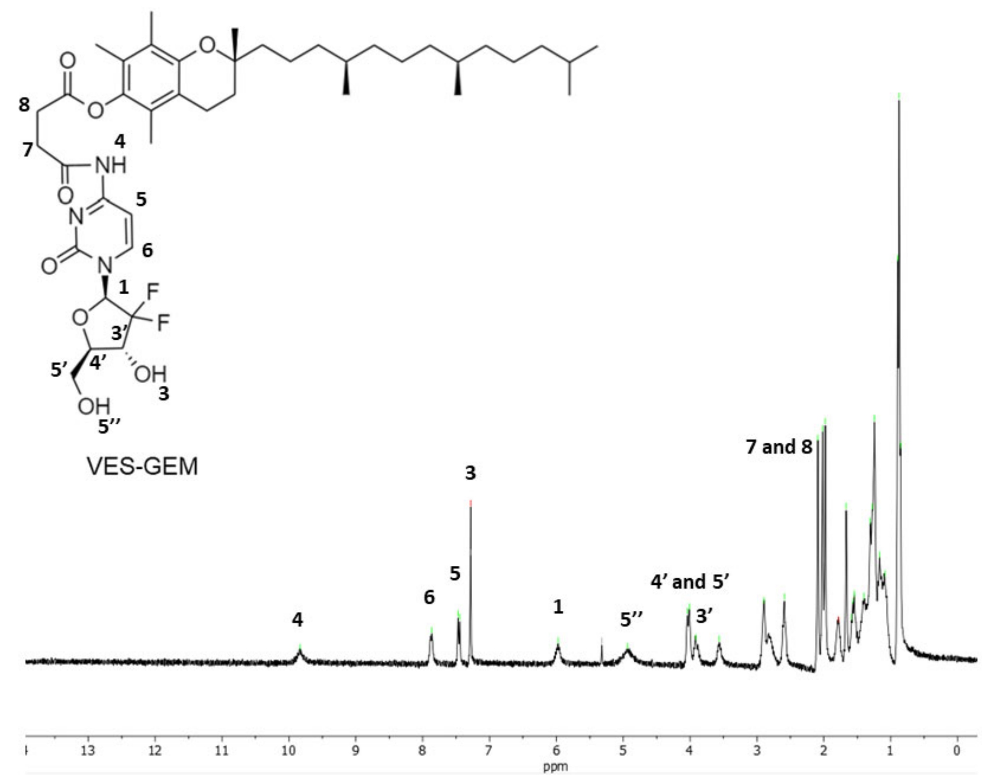

Figure S7. ^{1}H NMR of VES-GEM.

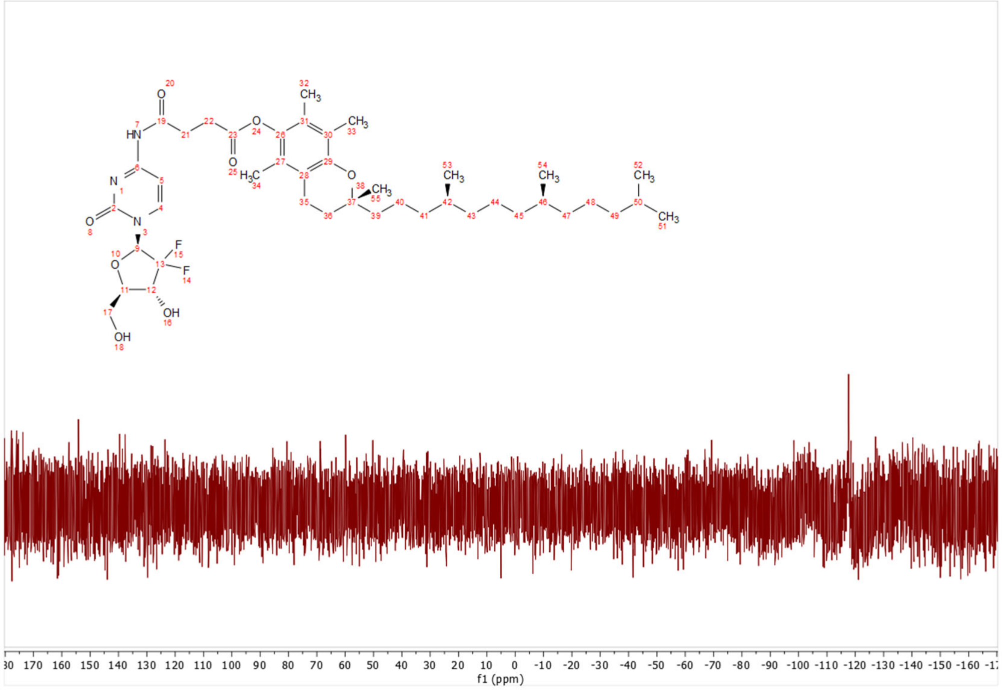

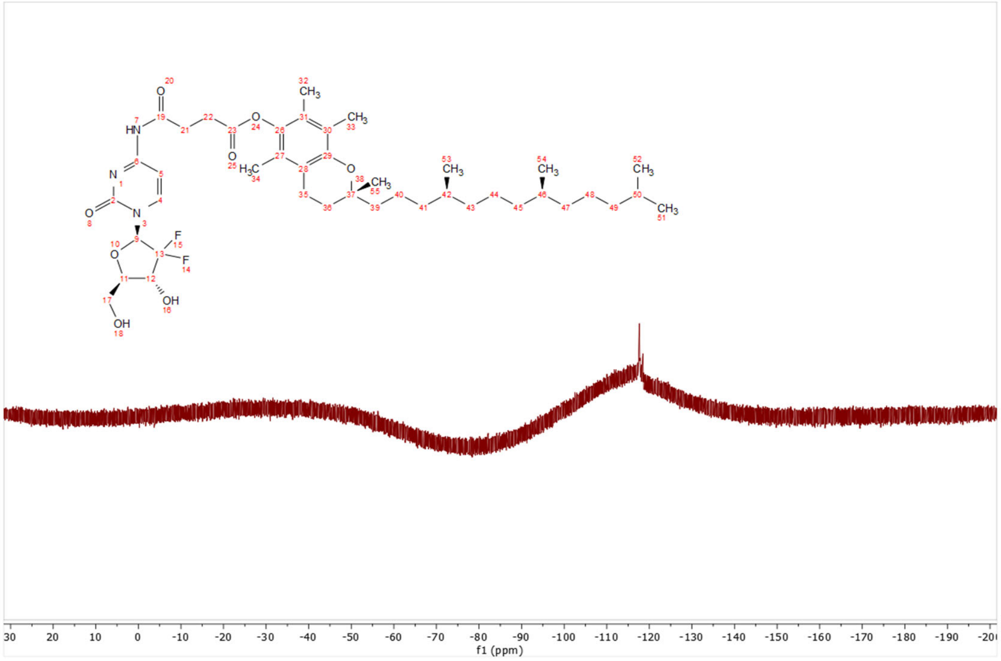

Figure S8. ^{19}F NMR of VES-GEM.

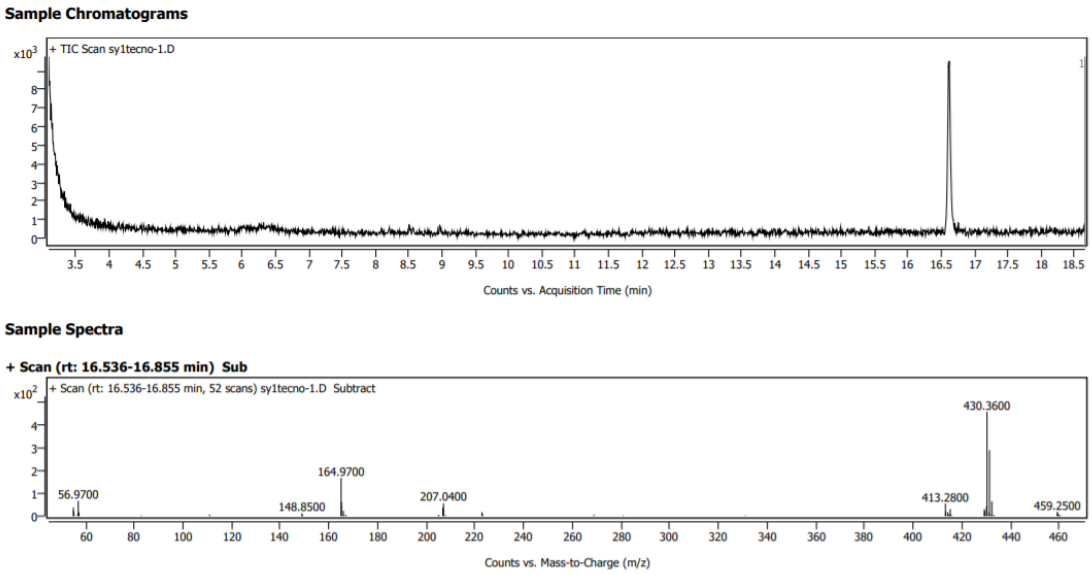

Figure S9. MS of VES-GEM.

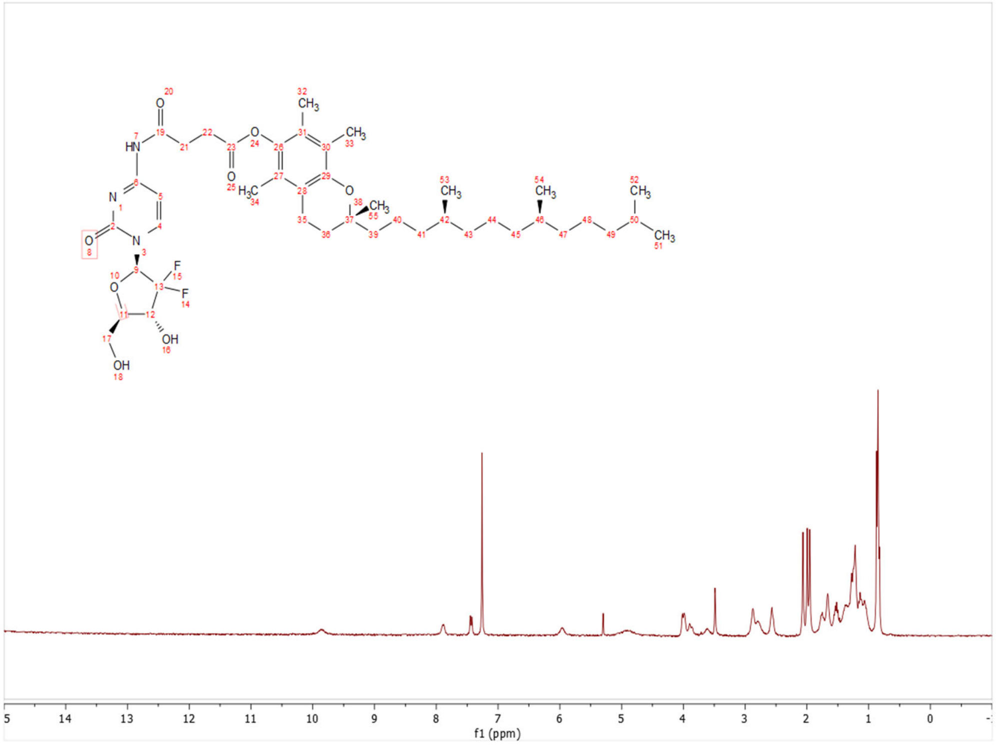

Figure S10. ^{1}H NMR of VES-GEM for stability assay.

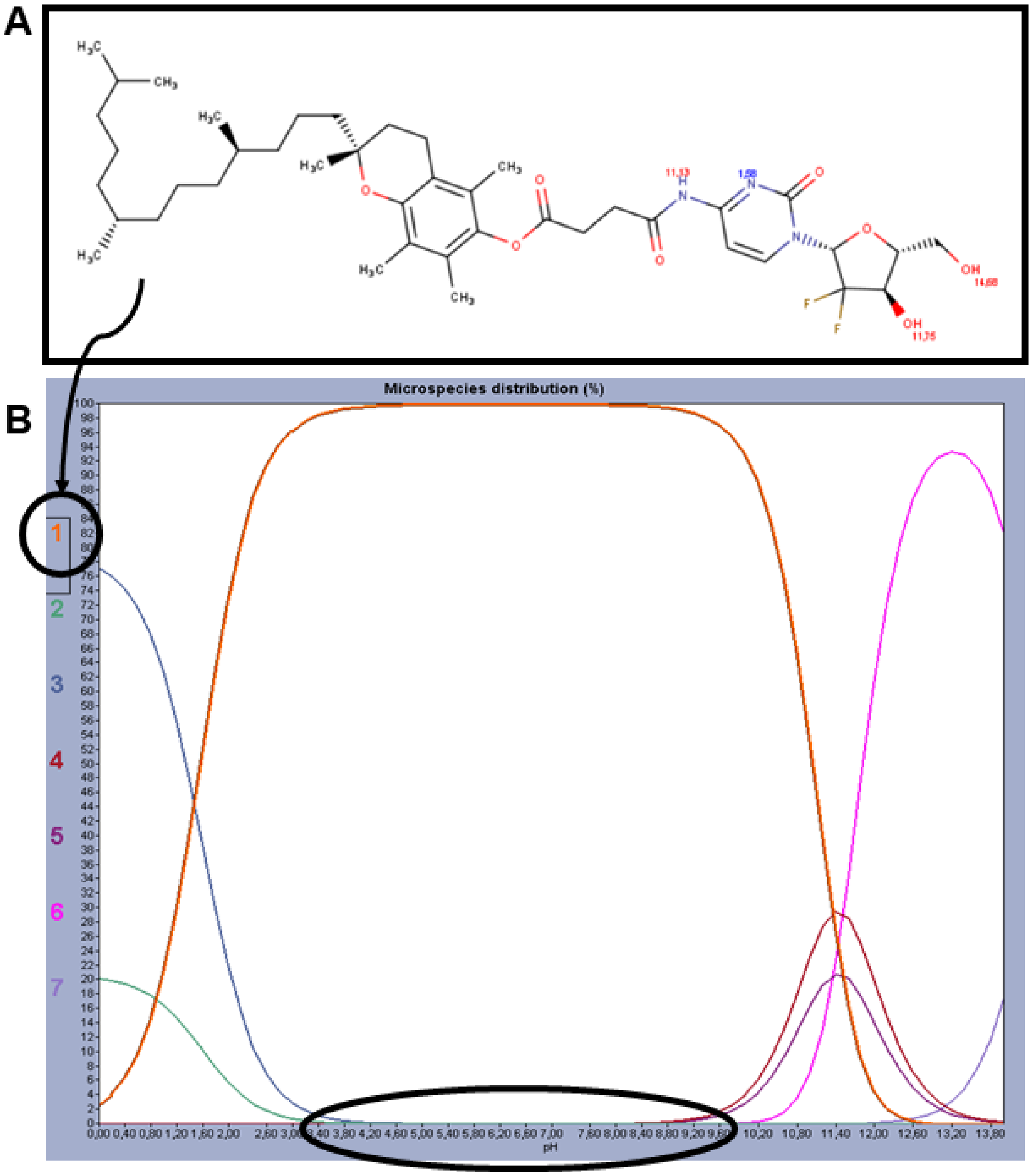

Figure S11. A) Chemical structure of the predominant VES-GEM conjugate form (#1) for pH 4-9. In red, numbered, pKa values for (-OH) and (-N) groups. B) microspecies distribution (%) as a function of pH (0-14). Chemicalize and MarvinView software models.

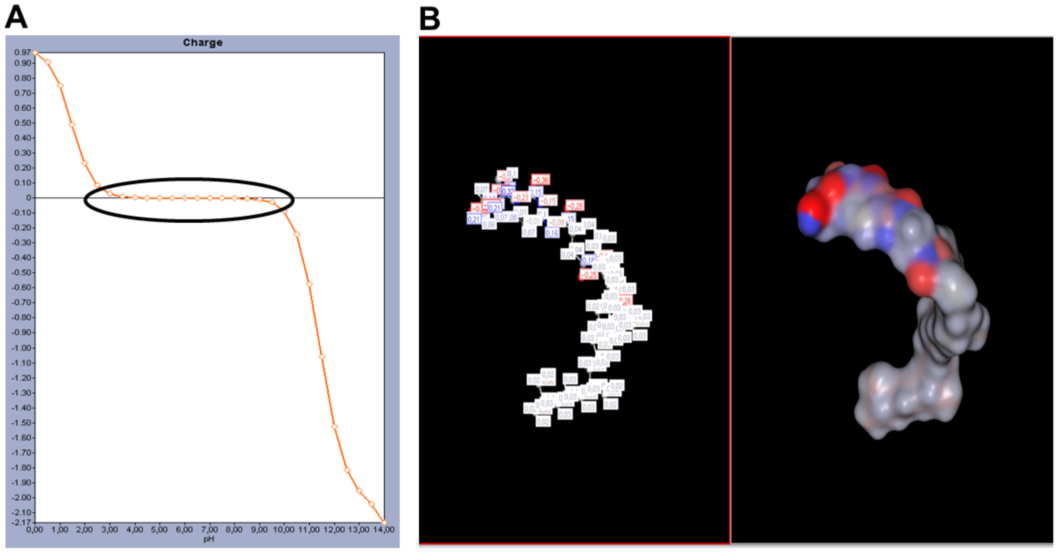

Figure S12. A) Chemical structure of the predominant VES-GEM conjugate form (#1) for pH 4-9. In red, numbered, pKa values for (-OH) and (-N) groups. B) microspecies distribution (%) as a function of pH (0-14). Chemicalize and MarvinView software models.

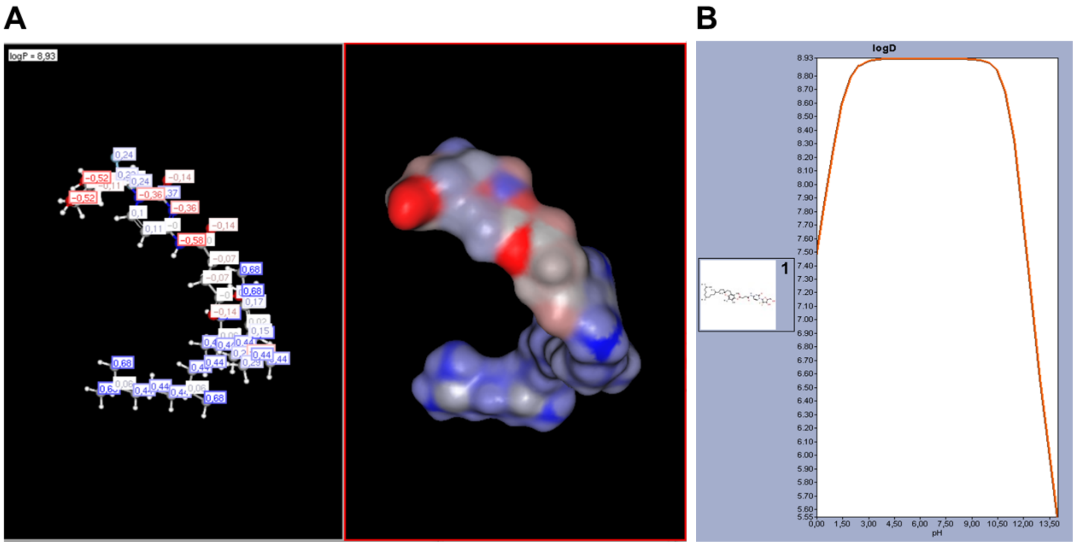

Figure S13. A) logP calculation for VES-GEM conjugate. B) LogD variation as a function of pH (0-14), Chemicalize and MarvinView software models.

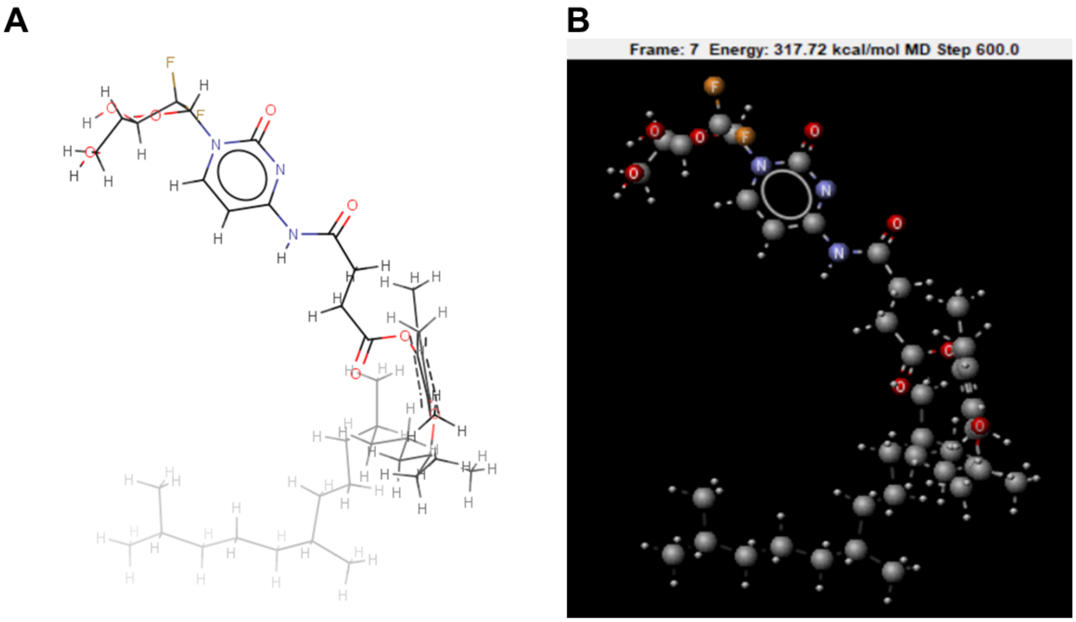

Figure S14. A) predicted 3D structure for VES-GEM conjugate. B) video animation capture of predicted 3D structure for VES-GEM conjugate. Chemicalize and MarvinView software models.

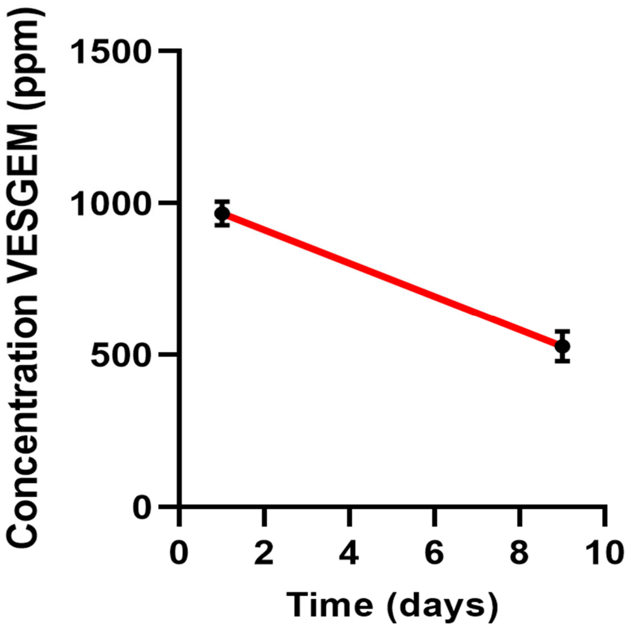

Figure S15. Stability at RT of VES-GEM stock solution in ethanol. A 800 ppm stock solution of VES-GEM was prepared in ethanol and left at RT and protected from light for 8 days. Aliquots were collected at time=1 day and time=8 day, diluted in ethanol 1:10, filtered and VES-GEM content measured through HPLC.

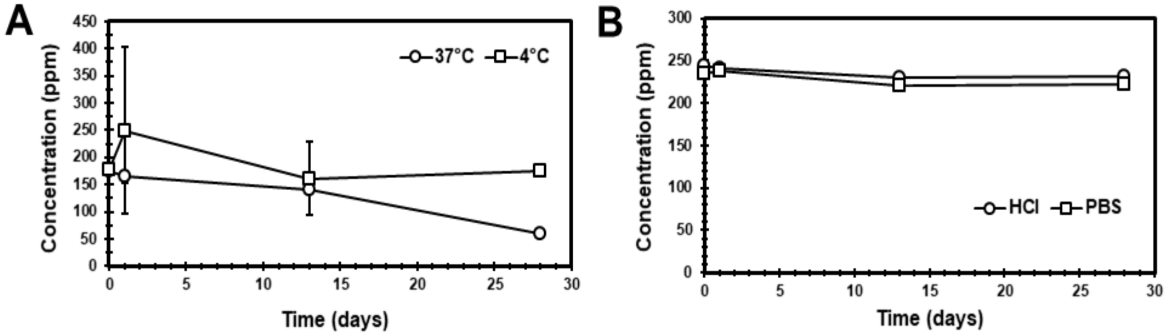

Figure S16. Stability of VES-GEM in ethanol pH=5 as a function of temperature (A) and GEM in acidic aqueous milieu (HCl or PBS pH=5, RT) (B).

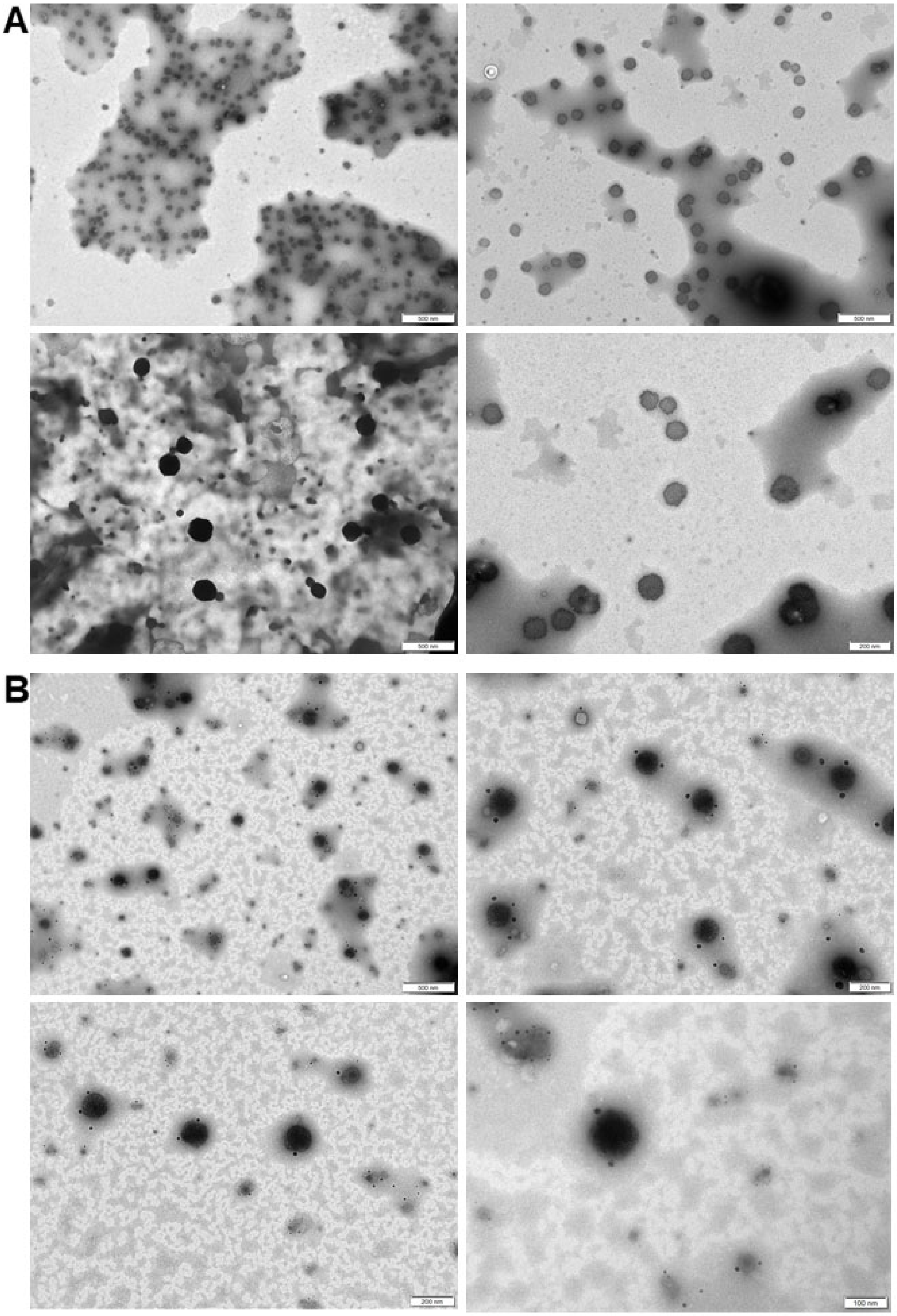

Figure S17. TEM picture of blank Pluronic® F68 micelles (0.86 %w/v) in PBS:water 50:50 v/v undiluted (A) and diluted 1:4 (B).

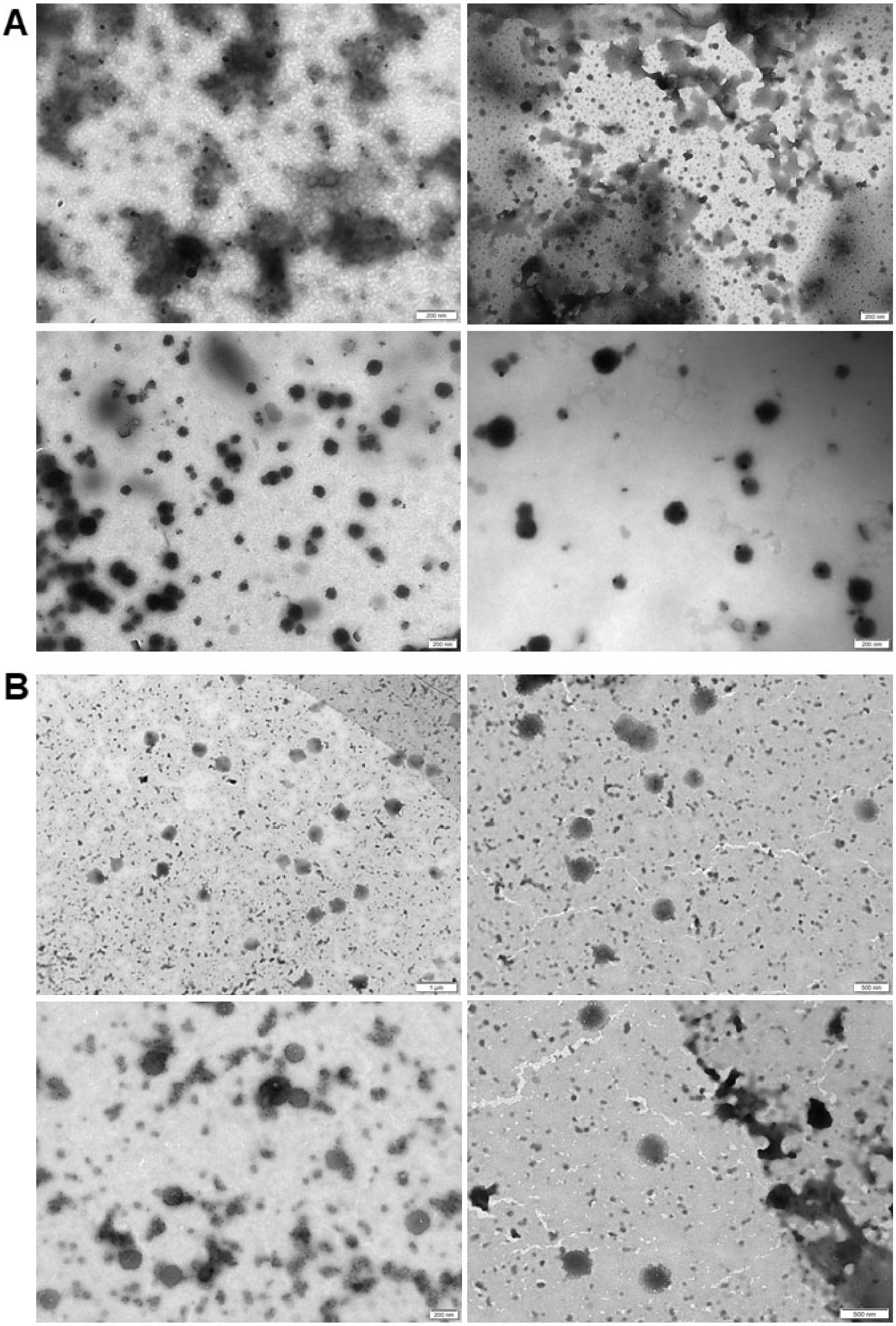

Figure S18. TEM picture of blank Pluronic® F127 (0.65 %w/v) micelles undiluted (A) and diluted 1:4 (B) in PBS:water 50:50 v/v.

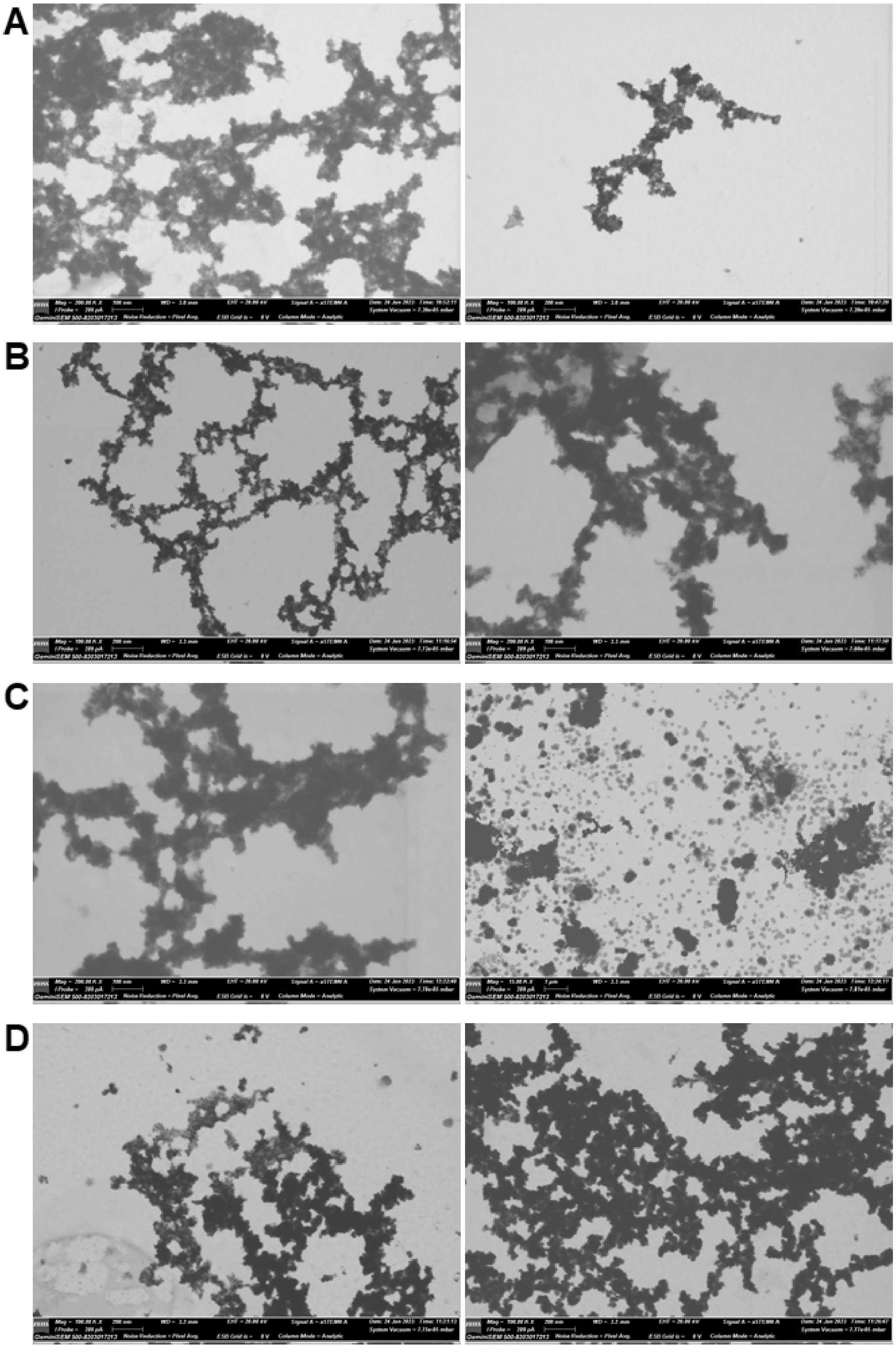

Figure S19. TEM picture of Pluronic® F68/VES-GEM (3/1) conjugate micelles (0.86 %w/v F68) undiluted (A), diluted 1:4 (B); Pluronic® F127/VES-GEM (1.5/1) conjugate micelles (0.65 %w/v F127) undiluted (C), diluted 1:4 (B), in PBS:water 50:50 v/v.

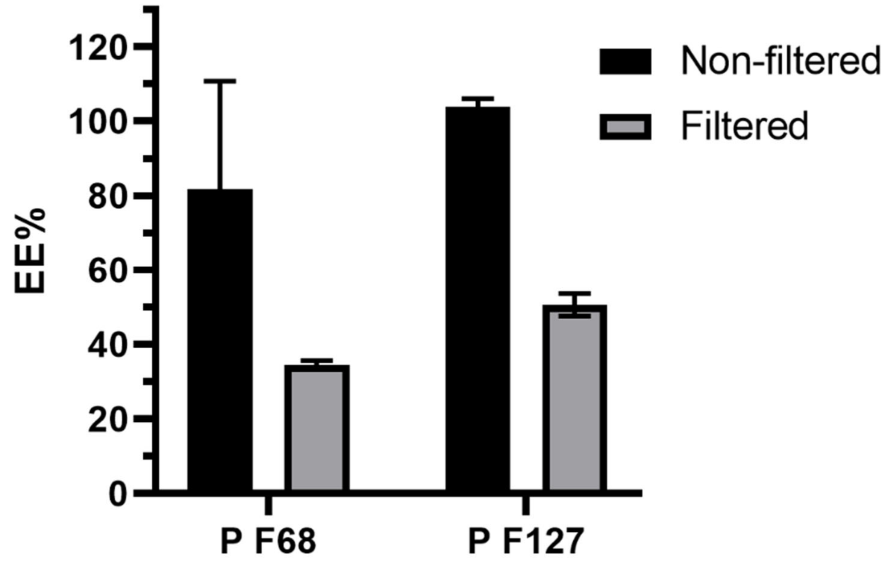

Figure S20. Encapsulation efficiency of non-filtered and filtered (hydrophilic PTFE syringe filter (25 mm, 0.4 µm)) of Pluronic® F68/VES-GEM (3/1) conjugate micelles (0.86 %w/v F68) and Pluronic® F127/VES-GEM (1.5/1) conjugate micelles (0.65 %w/v F127).

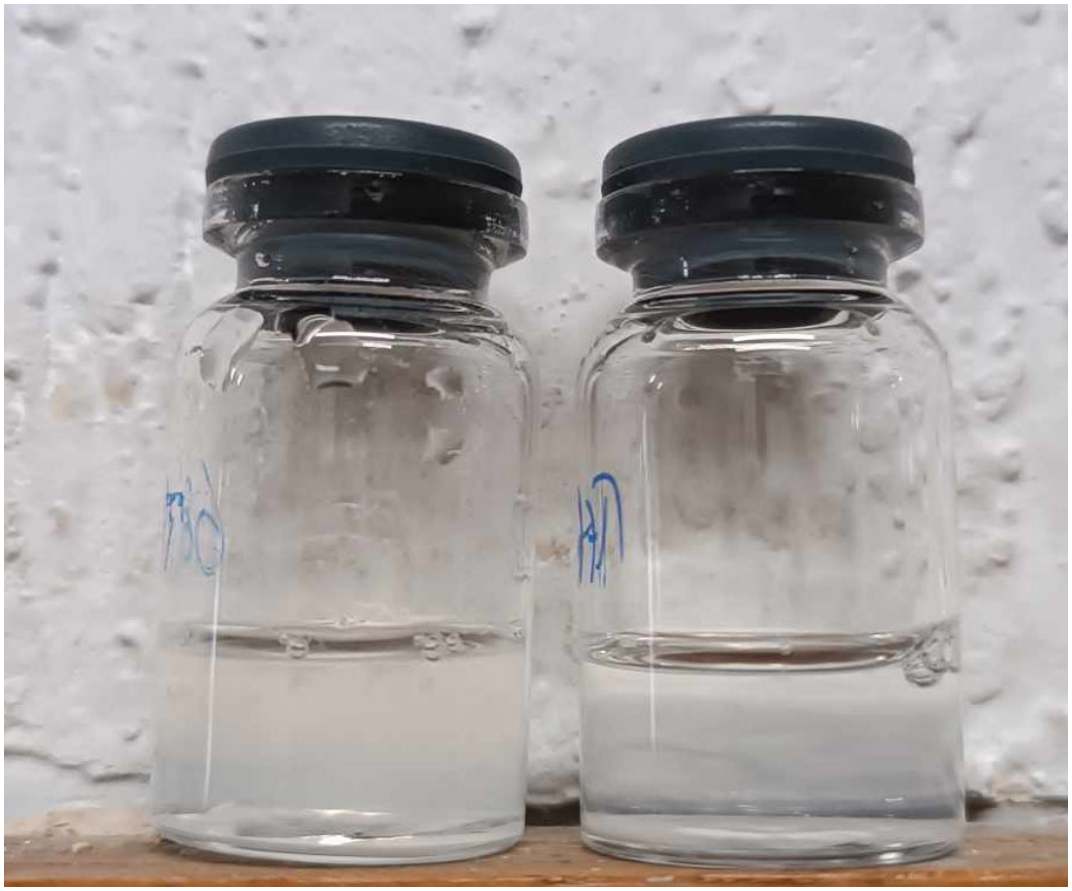

Figure S21. Appearance of Pluronic® F68/VES-GEM (15/1) conjugate micelles (4.3 % w/v F68) and Pluronic® F127/VES-GEM (7.5/1) conjugate micelles (3.25 %w/v F127).

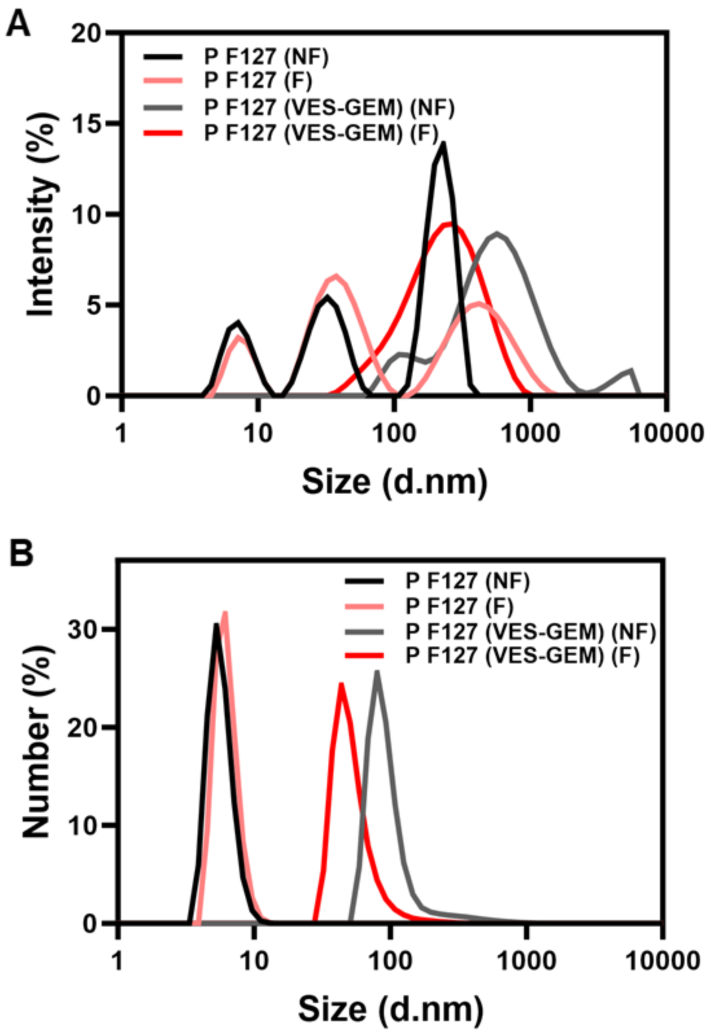

Figure S22. Intensity mode (%) (A) and number mode (%) (B) size distribution of filtered and non-filtered Pluronic® F68, Pluronic® F68/VES-GEM (3/1), Pluronic® F127 and Pluronic® F127/VES-GEM (1.5/1) micelles in DLS, after ultrasonication 36 s (6 s on, 3 s off).

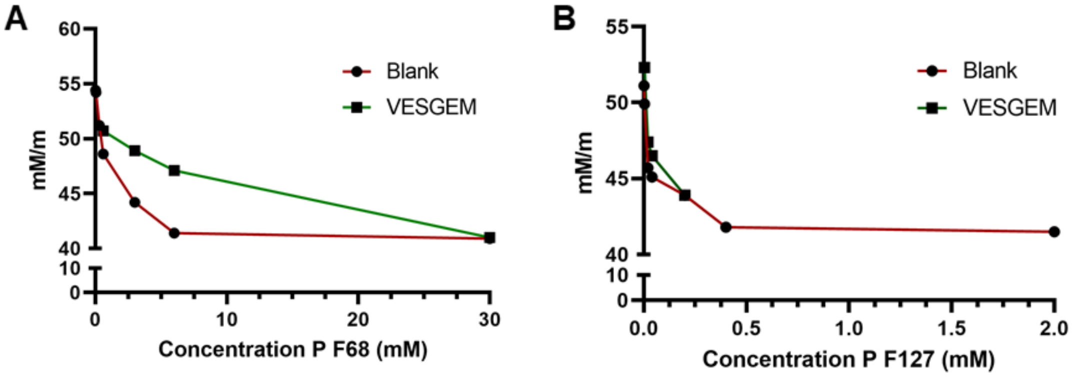

Figure S23. Influence of VES-GEM loading in the CMC of Pluronic® F68/VES-GEM (A) and Pluronic® F127/VES-GEM conjugate micelles (B) in PBS: water 50:50 v/v. Data plotted as surface tension (mM/m) versus concentration of surfactant, at RT. Abbreviations: P F68 - Pluronic® F68 micelles; P F127 - Pluronic® F127 blank micelles.

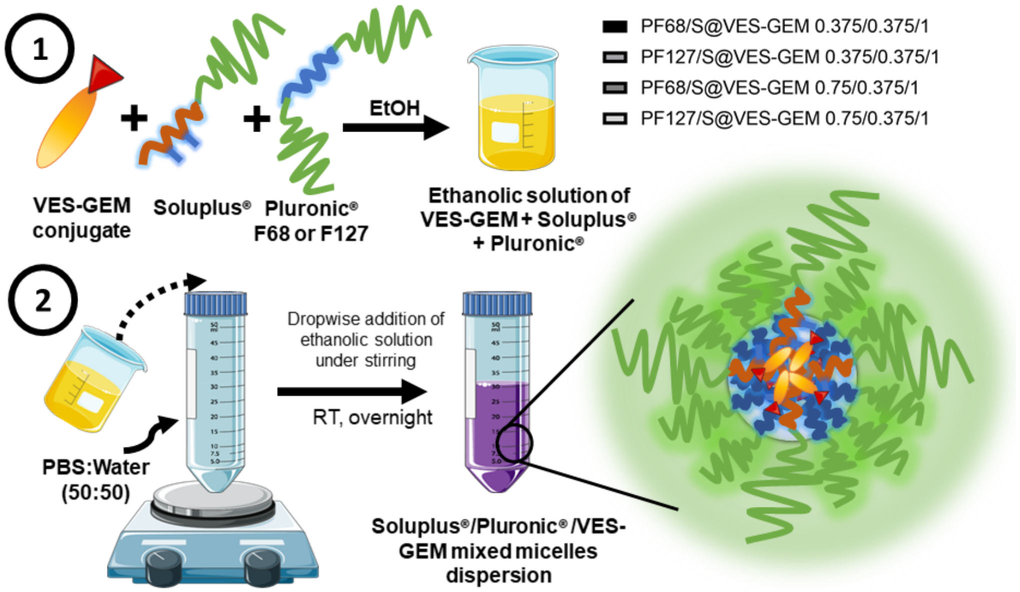

Figure S24. Schematic illustration o the preparation of Pluronic®/Soluplus®@VES-GEM micelles through solvent evaporation method. Soluplus® concentration was fixed at 14.72 mg/mL.

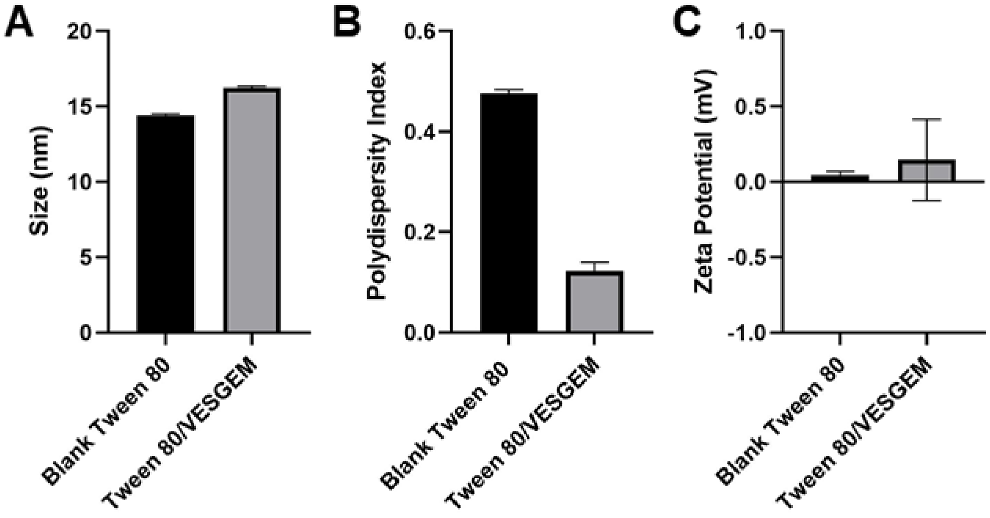

Figure S25. (A) Size, (B) PDI and (C) ZP of Tween 80/VES-GEM micelles.

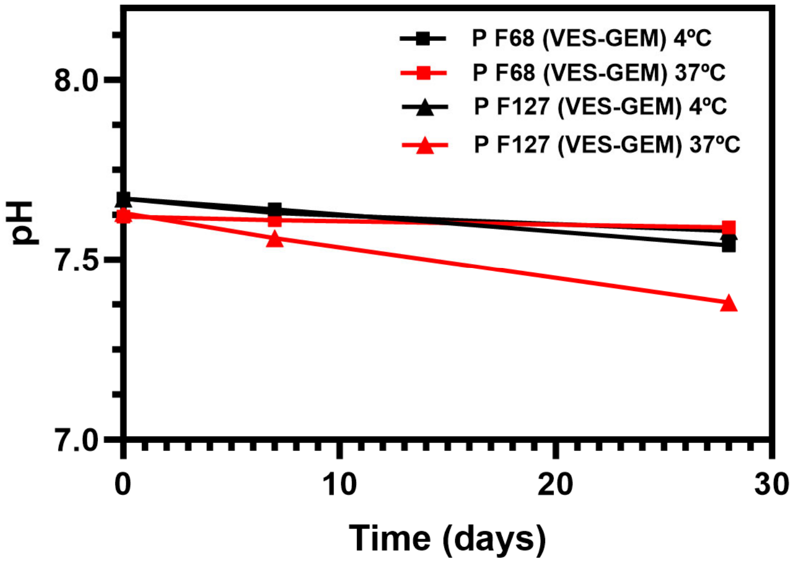

Figure S26. pH measurement at 4ºC and 37ºC of Pluronic® F68/VES-GEM (3/1) and Pluronic® F127/VES-GEM (1.5/1) micelles, PBS: water 50:50 v/v.

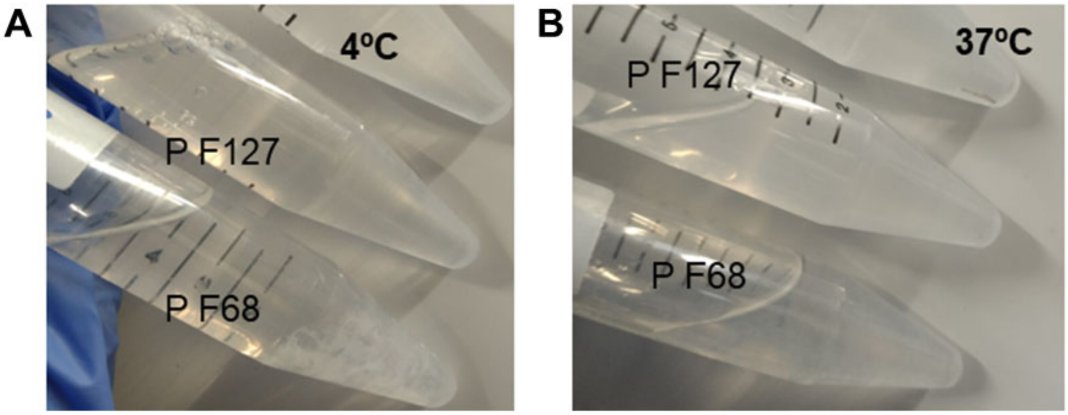

Figure S27. Appearance of Pluronic® F68/VES-GEM (3/1) and Pluronic® F127/VES-GEM (1.5/1) conjugates micelles after 2 days at 4ºC (A) and 37ºC (B). Abbreviations: P F68 - Pluronic® F68/VES-GEM (3/1) micelles; P F127 - Pluronic® F127/VES-GEM (1.5/1) micelles.

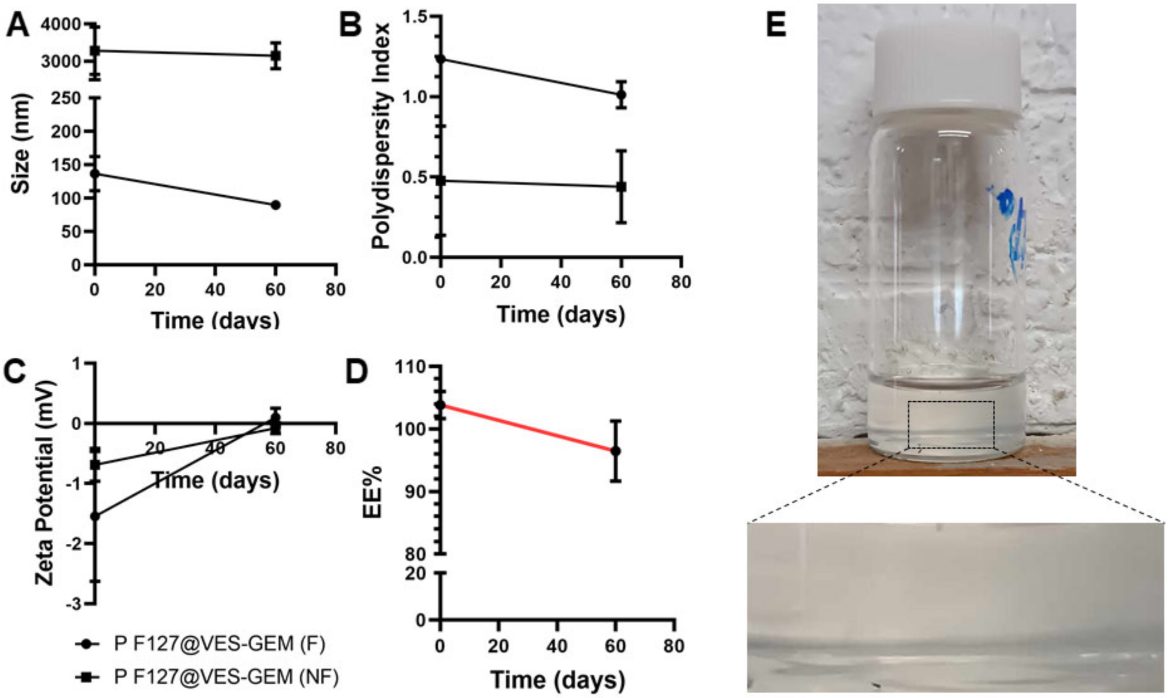

Figure S28. (A) Size, (B) PDI, (C) ZP, (D) EE and (E) physical appearance of Pluronic® F127/VES-GEM (1.5/1) conjugate micelles.

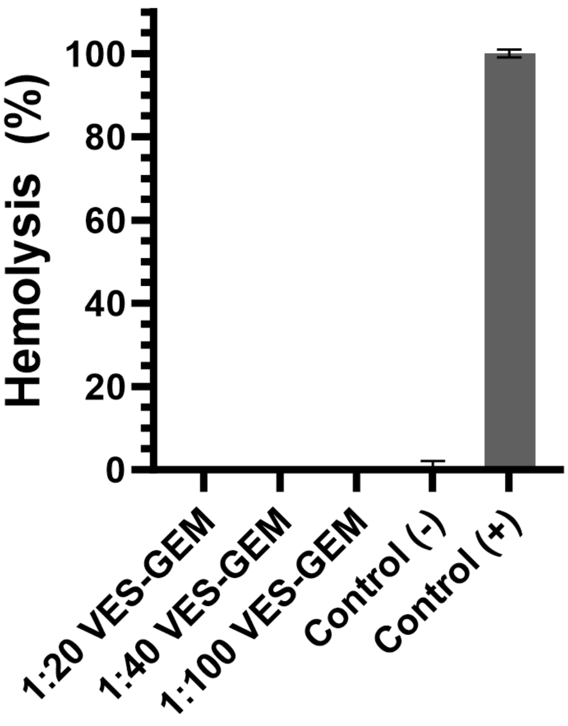

Figure S29. Haemolysis rate (%) 1:20, 1:40, 1:100 dilution of VES-GEM stock solution 1 mg/mL, negative control (PBS) and positive control (DMSO).

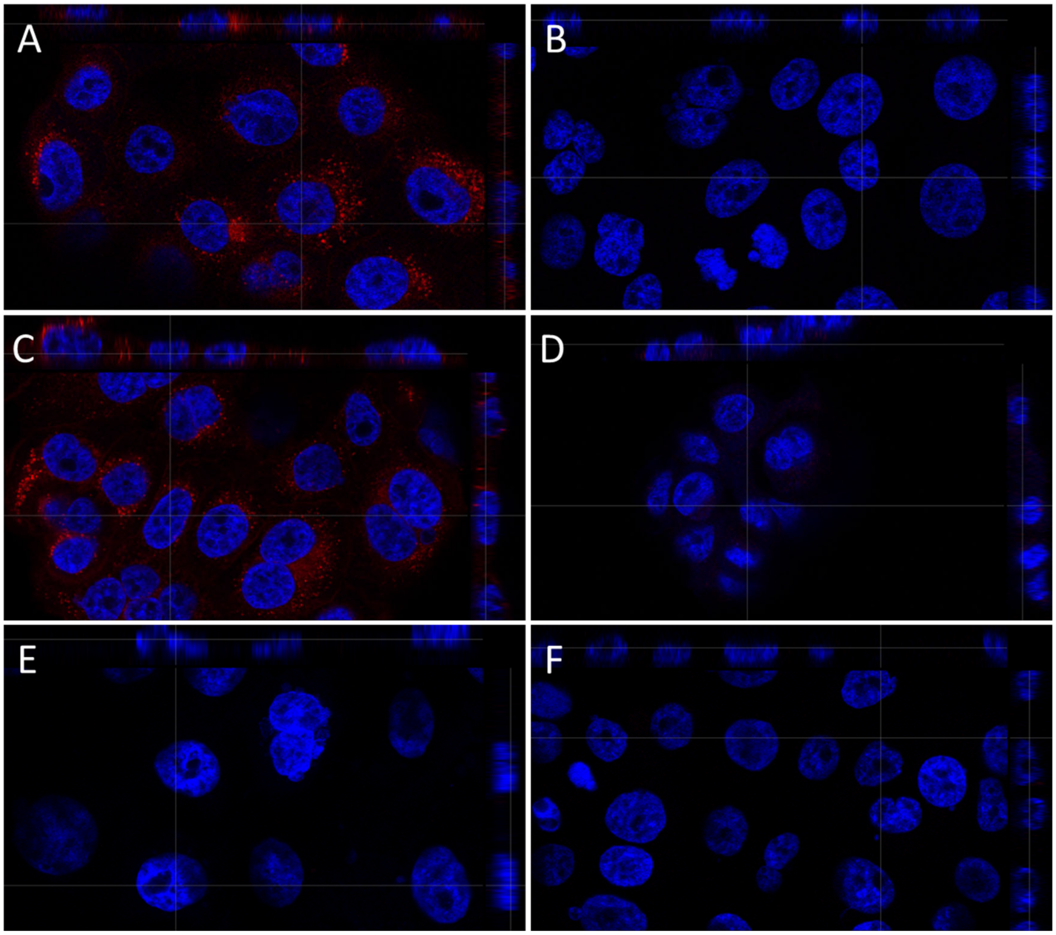

Figure S30. Confocal images representing the X−Y, X−Z, and Y−Z planes of BxCP3 cells incubated in the presence Pluronic® F68/VES-GEM (A,B) and Pluronic® F127/VES-GEM (C,D) micelles loaded with Nile red (A.,C) or blank (B, D), and controls treated with free Nile red (E) or culture medium (F). Cell nuclei were stained with DAPI in blue and Nile red is shown in red. X axis length is 136 μm.

Table S1. Size and PDI of filtered (hydrophilic PTFE syringe filter (25 mm, 0.4 µm)) and non-filtered Pluronic® F68/VES-GEM (15/1) and Pluronic® F127/VES-GEM (7.5/1) micelles.

Formulations	Filtered	Size (nm) ± S.D.	PDI ± S.D.
Pluronic® F68/VES-GEM (15/1)	No	1488.33 ± 163.27	0.491 ± 0.273
Pluronic® F68/VES-GEM (15/1)	Yes	1912.00 ± 365.68	0.303 ± 0.270
Pluronic® F127/VES-GEM (7.5/1)	No	639.67 ± 147.89	1.480 ± 0.166
Pluronic® F127/VES-GEM (7.5/1)	Yes	435.04 ± 61.70	1.640 ± 0.134

Table S2. Size, ZP and PDI of filtered and non-filtered Pluronic® F68/VES-GEM and Pluronic® F127/VES-GEM micelles subjected to 15 min of sonication, RT.

Formulations	Molar ratio	Sonication time	Filtered before measurement	Size ± S.D.	PDI ± S.D.
Pluronic® F68/VES-GEM	3/1	15 min	Yes	1128.67 ± 81.98	1.328 ± 0.024
			No	2547.67 ± 686.35	0.905 ± 0.126
Pluronic® F127/VES-GEM	1.5/1		Yes	284.37 ± 153.11	1.175 ± 0.141
			No	2305.67 ± 223.64	0.485 ± 0.106

Table S3. Size, ZP and PDI of filtered and non-filtered Pluronic® F68/VES-GEM and Pluronic® F127/VES-GEM micelles subjected to 6 s of ultrasonication (RT, 10% amplitude).

Formulations	Molar ratio	Ultrasonication time	Filtered before measurement	Size ± S.D.	PDI ± S.D.
Pluronic® F68/VES-GEM	3/1	6 s	Yes	280.37 ± 13.48	0.741 ± 0.099
			No	2356 ± 204.14	0.852 ± 0.160
Pluronic® F127/VES-GEM	1.5/1		Yes	309.90 ± 0.294	0.265 ± 0.003
			No	621.17 ± 22.95	0.400 ± 0.080
